# Clinical and Epidemiological Profile of Atrial Fibrillation in a High-Altitude Tertiary-Care Setting

**DOI:** 10.3390/medicina62091736

**Published:** 2026-09-09

**Authors:** Vladimir Ernesto Ullauri-Solórzano, René Antonio Vicuña Mariño, Diego Ricardo Egas Proaño, Diana Moreira-Vera, Henrry Oswaldo Jaramillo Prado, Ana Gabriela Finke Barriga, Gabriela Tatiana León Molina, Juan José Paz y Miño, Ronald Alfredo Cevallos Macías, Jorge Vasconez-Gonzalez, Juan S. Izquierdo-Condoy, Esteban Ortiz-Prado

**Affiliations:** 1 Hospital Metropolitano de Quito, Quito 170129, Ecuador; 2 One Health Research Group, Universidad de las Americas, Quito 170137, Ecuador

**Keywords:** atrial fibrillation, anticoagulation, high altitude, Latin America, hospital epidemiology

## Abstract

*Background and Objectives*: Atrial fibrillation (AF) is the most common sustained cardiac arrhythmia and a major cause of stroke, heart failure, and death. Evidence on AF in Latin American and Andean tertiary-care settings remains limited. The objective was to describe the demographic and clinical profile, documented thromboembolic and bleeding risk, in-hospital management, and outcomes of adults with AF treated at a tertiary hospital located at 2850 m above sea level. *Materials and Methods*: We retrospectively reviewed unique adult patient records from January 2021 through December 2023 identified by ICD-10 code I48 and explicit clinician documentation of AF. Independent electrocardiographic adjudication was not performed. Demographics, comorbidities, chart-recorded AF category, risk scores, echocardiographic findings, in-hospital treatments, and outcomes were summarized descriptively. *Results*: Among 238 patients, mean age was 77.9 ± 13.3 years, 55.0% were male, and 97.1% were recorded as mestizo. Hypertension (58.0%) and heart failure (22.7%) were the most frequent comorbidities. CHA_2_DS_2_-VASc and HAS-BLED scores were documented for 199 patients (83.6%); their respective means were 3.3 ± 1.4 and 2.3 ± 1.1. Any anticoagulant was administered during hospitalization to 195 patients (81.9%), but previous and discharge therapy, indications, contraindications, dosing, and temporary interruptions were not consistently available. Five patients died in hospital (2.1%). Secondary exploratory comparisons by survival status were hypothesis-generating, and none remained significant after false-discovery-rate correction. *Conclusions*: This cohort describes an older, comorbid population treated at a hospital situated at high altitude. The study does not establish an altitude-related AF phenotype, the appropriateness of chronic anticoagulation, or prognostic factors for mortality. Prospective multicentre studies with adjudicated AF, individual altitude exposure, oxygenation and haematologic measures, and longitudinal treatment data are warranted.

## 1. Introduction

Atrial fibrillation (AF) is the most prevalent sustained cardiac arrhythmia worldwide, characterized by irregular and often rapid atrial activation that impairs cardiac output and predisposes to thromboembolism [1,2]. It is a leading cardiac cause of ischaemic stroke, accounting for approximately 15% of all cases, and its risk rises exponentially with age, roughly doubling each decade from 0.55% at 50–59 years to about 9% at 80–89 years [3,4].

Beyond its clinical burden, AF imposes a substantial economic impact, with AF-related hospitalization costs in the United States estimated at USD 6–26 billion annually and incremental direct costs of roughly USD 28,000 per patient [5,6,7]. The global prevalence of AF was estimated at 37.6 million cases in 2017 and is projected to continue rising, reinforcing its status as a global epidemic [8,9,10,11,12].

In Latin America, data from regional cohorts estimate an AF prevalence of approximately 2.4% among adults older than 40 years [12]. Evidence from Ecuador remains limited: national burden-of-disease estimates reported thousands of AF-related hospital discharges and deaths with substantial years of life lost [12], a 25% AF prevalence among mestizo patients with poorly controlled type 2 diabetes [8,13], and rheumatic mitral valve disease, hypertension, and idiopathic AF as leading local etiologies [14].

An additional, understudied dimension is altitude. Approximately 6.6% of the world’s population lives above 1500 m, and Quito, Ecuador’s capital, is located at about 2850 m above sea level [15]. Chronic exposure to hypobaric hypoxia can influence sympathetic activity and pulmonary vascular physiology [16,17]. However, a retrospective hospital-based study conducted at a single altitude, without individual exposure measurements or a lower-altitude comparator, cannot determine whether altitude influenced AF occurrence or presentation. Accordingly, altitude was treated as contextual information rather than as an individual exposure or causal determinant, consistent with recent descriptive studies of heart failure and acute coronary syndrome conducted at the same Andean tertiary hospital [18,19].

Against this background, the clinical and epidemiological characteristics of AF in Ecuador—and in high-altitude care settings generally—remain underexplored. This study aimed to describe the demographic and clinical profile, documented thromboembolic and bleeding risk, in-hospital management, and outcomes of adults with AF treated at a tertiary-care hospital located at high altitude between 2021 and 2023.

## 2. Materials and Methods

### 2.1. Study Design

We conducted a retrospective observational study based on anonymized medical records from the Hospital Metropolitano de Quito, Ecuador. The analysis was designed to characterize the cohort and inpatient care rather than to estimate causal effects or define an altitude-specific AF phenotype.

### 2.2. Setting

The Hospital Metropolitano de Quito is a tertiary care centre serving a predominantly private-sector population in Quito, the capital of Ecuador, located at approximately 2850 m above sea level [20]. Hospital altitude is reported as contextual information only. Individual residential altitude, duration of residence, oxygen saturation, haematologic adaptation, and prior lower-altitude exposure were not available; consequently, altitude was not analyzed as an individual exposure.

### 2.3. Participants, Sampling, and Eligibility

Consecutive records were screened for the prespecified period January 2021 through December 2023, which represented the closed dataset available under the approved retrospective protocol at the time of extraction. Records after 2023 were outside the approved analytic window. Eligible patients were aged ≥ 18 years and had ICD-10 code I48 together with explicit AF documentation by the treating clinical team. The unit of analysis was the patient, and each individual contributed one record. Source electrocardiographic tracings were not systematically available for independent re-review; therefore, case ascertainment relied on clinician documentation. The extract did not permit complete exclusion of atrial flutter when coding or narrative documentation was ambiguous, nor consistent distinction of AF as a primary, secondary, or incidental diagnosis. Records with insufficient clinical information, outside the study period, or labelled only as unspecified arrhythmia were excluded. Of 246 records screened, eight were excluded for insufficient data, yielding 238 patients.

### 2.4. Variables

We abstracted sex, age, residence, recorded ethnicity, anthropometric measures, comorbidities, AF presentation, symptoms, chart-recorded AF category, family history of AF, inpatient pharmacological and non-pharmacological treatment, echocardiographic findings, and outcomes. “Regular physical activity” was defined operationally as habitual exercise or physical activity explicitly documented in the chart; no standardized duration or intensity threshold was available. CHA_2_DS_2_-VASc and HAS-BLED were analyzed as recorded by the treating team. The 2024 ESC guideline now favours CHA_2_DS_2_-VA, treating female sex as a modifier rather than an independent point; a valid retrospective recalculation was not possible because patient-level score components were not retained in the de-identified aggregate export [21]. The primary outcome was in-hospital mortality.

### 2.5. Statistical Analysis

Categorical variables are summarized as frequency and percentage and continuous variables as mean ± standard deviation. Denominators are reported when they differed from the full cohort. Missing observations were handled by available-case analysis without imputation. Because only five in-hospital deaths occurred, comparisons between survivors and non-survivors were considered secondary, post hoc, and strictly hypothesis-generating. Continuous variables with available group sizes, means, and standard deviations were compared using Welch’s *t* test, and categorical variables using two-sided Fisher’s exact test. Mean differences and conditional maximum-likelihood odds ratios are reported with 95% confidence intervals. To address multiple comparisons, false-discovery-rate-adjusted q values were calculated using the Benjamini–Hochberg procedure across all 31 survival-status comparisons. No multivariable model was fitted because the number of events was insufficient for reliable, adjusted estimation. Analyses were conducted in IBM SPSS Statistics v29.0 and independently reproduced in Python version 3.12 using standard statistical libraries (pandas/SciPy) for verification.

### 2.6. Ethical Statement

The study adhered to the Declaration of Helsinki and was approved by the Ethics Committee for Research in Human Beings of the Universidad de las Américas (CEISH-UDLA), protocol 2024-OBS-020, which granted a waiver of informed consent given the retrospective, anonymized design. Patient confidentiality was protected through data anonymization in accordance with applicable Ecuadorian regulations.

## 3. Results

### 3.1. Demographic Characteristics and Medical History

**Figure 1:**
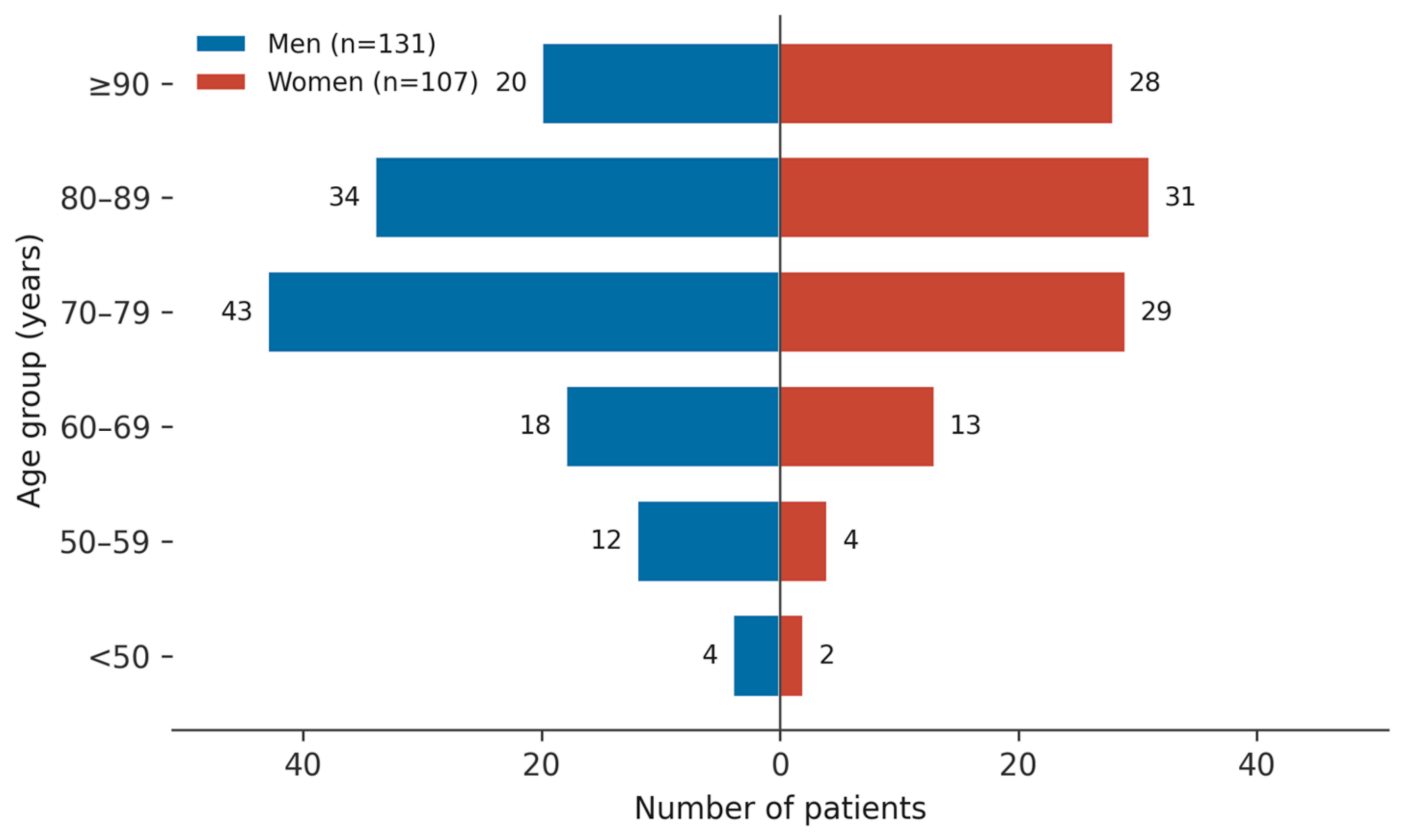
Age and sex distribution of the atrial fibrillation cohort (n = 238). The population was concentrated in the eighth and ninth decades of life; women predominated in the oldest age groups.

Of 246 records screened, eight were excluded for insufficient clinical information, leaving 238 unique patients. Mean age was 77.9 ± 13.3 years, 131 patients (55.0%) were male, and 231 (97.1%) were recorded as mestizo. Mean body-mass index was 27.6 ± 4.5 kg/m^2^, and 228 patients (95.8%) resided in Ecuador (Figure 1).

Chart-documented regular physical activity was present in 41 patients (17.2%), tobacco use in 44 (18.5%), and alcohol use in five (2.1%). Among patients with tobacco exposure, mean cumulative exposure was 20.1 ± 15.1 pack-years (Table 1).

At least one underlying medical condition was documented in 78.2% of the cohort. Hypertension was the most frequent comorbidity, affecting 138 patients (58.0%), followed by heart failure in 54 (22.7%), hypothyroidism in 45 (18.9%), type 2 diabetes mellitus in 36 (15.1%), dyslipidaemia in 34 (14.3%), and obesity in 32 (13.4%). Previous myocardial infarction was documented in 24 patients (10.1%), including five with previous ST-elevation myocardial infarction. Chronic kidney disease, previous ischaemic stroke, and sleep apnoea were less frequent (Table 2).

### 3.2. AF Presentation and In-Hospital Management

Overall, 121 patients (50.8%) had symptoms attributed to AF. Among symptomatic patients, palpitations were most frequent (68.0%), followed by dyspnoea (22.7%); other symptoms were uncommon.

**Figure 2:**
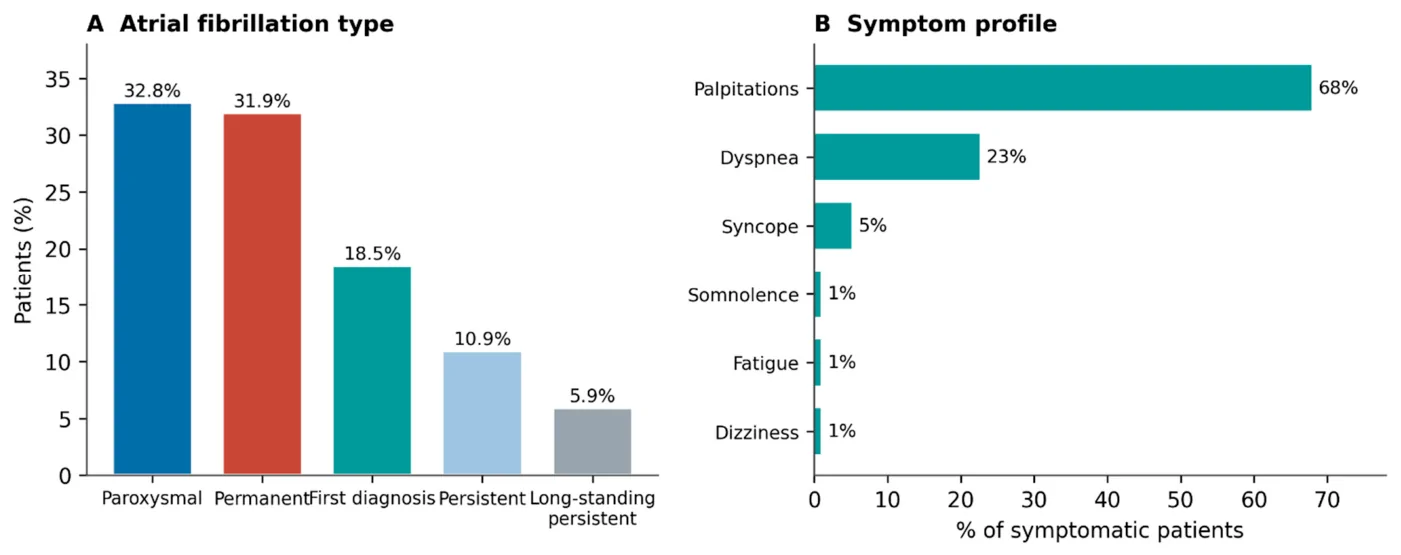
Chart-recorded atrial fibrillation category and symptom profile. (**A**) Distribution of atrial fibrillation types in the overall cohort. (**B**) Frequency of reported symptoms among patients with symptomatic atrial fibrillation.

The source database used mutually exclusive chart-recorded categories: paroxysmal (32.8%), permanent (31.9%), first-diagnosed (18.5%), persistent (10.9%), and long-standing persistent AF (5.9%). “First-diagnosed” describes presentation status rather than a temporal AF pattern and was retained only when an established temporal subtype was not documented. Family history refers specifically to AF and was recorded in 14 patients (5.9%) (Figure 2).

At admission, 157 patients (66.0%) were not in sinus rhythm, 98 (41.2%) were classified as New York Heart Association functional class II, and 182 (76.5%) had pharmacological AF treatment documented. Beta-blockers were the most frequent rate-control agents and amiodarone the most frequent rhythm-control medication. Mean hospital stay was 4.9 ± 4.6 days (Table 3).

### 3.3. Documented Thromboembolic and Bleeding Risk and In-Hospital Anticoagulant Administration

CHA_2_DS_2_-VASc and HAS-BLED were formally documented for 199 of 238 patients (83.6%); 39 patients (16.4%) lacked a formal charted score. Mean CHA_2_DS_2_-VASc was 3.3 ± 1.4 (median 3, IQR 2–4), and 185 scored patients (93.0%) had a value ≥ 2. These values describe scores documented during 2021–2023 and should not be interpreted as recalculated CHA_2_DS_2_-VA eligibility under the 2024 ESC guideline [21].

Mean HAS-BLED was 2.3 ± 1.1 (median 2, IQR 2–3); 65 scored patients (32.7%) had a value ≥3. Formal scores were missing when they were not documented in the chart, and component-level data were insufficient for validated retrospective reconstruction (Table 4).

During hospitalization, 195 patients (81.9%) received at least one anticoagulant. Among the 185 patients with a charted CHA_2_DS_2_-VASc score ≥ 2, 161 (87.0%) received an anticoagulant and 24 (13.0%) did not. Enoxaparin accounted for 43 of 195 treated patients (22.1%). These data capture administration during the index hospitalization only and do not establish chronic stroke-prevention therapy.

### 3.4. Echocardiographic Findings

Available-case echocardiographic summaries showed a mean left-ventricular ejection fraction of 59.2 ± 11.7%, left-atrial dilation in 99 of 233 patients with recorded status (42.5%), and a mean left-atrial volume index of 51.9 ± 25.3 mL/m^2^.

Mean estimated systolic pulmonary artery pressure was 41.9 ± 16.8 mmHg and mean E/e′ ratio was 12.5 ± 6.0. Tricuspid and mitral valve abnormalities were documented in 104 (43.7%) and 81 (34.0%) patients, respectively; wall-motion abnormalities were present in 25 (10.5%) (Table 5).

### 3.5. In-Hospital Mortality and Exploratory Survival-Status Comparisons

Five patients died during hospitalization, corresponding to an in-hospital mortality of 2.1%. In secondary exploratory comparisons, non-survivors were older and had a lower mean body-mass index than survivors. Absence of a documented ECG at admission and absence of in-hospital anticoagulant administration also yielded nominal between-group differences. However, none of the 31 comparisons remained statistically significant after false-discovery-rate correction (all q > 0.05; Supplementary Table S1). Given the small number of deaths, wide confidence intervals, and multiple comparisons, these findings should be interpreted exclusively as hypothesis-generating and not as evidence of independent prognostic effects.

## 4. Discussion

This retrospective study describes 238 adults with AF treated at a tertiary hospital situated at 2850 m above sea level. The cohort was predominantly elderly and had a substantial cardiovascular comorbidity burden. Risk scores were frequently high when documented, any anticoagulant was commonly administered during hospitalization, and in-hospital mortality was 2.1%. These observations characterize hospital practice; they do not define a high-altitude AF phenotype, establish treatment appropriateness, or support prognostic conclusions.

Advanced age, hypertension, heart failure, diabetes, obesity, chronic kidney disease, sleep-disordered breathing, tobacco exposure, and alcohol use are recognized AF determinants and targets of comprehensive risk-factor management [21,22,23]. The mean age of 77.9 years and the predominance of hypertension and heart failure in this cohort are consistent with that framework. Contemporary evidence also emphasizes important variation in AF burden, diagnostic access, and management across the Americas, with persistent evidence gaps in Latin American and Indigenous populations [23].

The chart-recorded AF categories require caution. First-diagnosed AF is not mutually exclusive with paroxysmal, persistent, or permanent patterns in guideline terminology; in this dataset it was used only when an established temporal subtype was not documented. This limitation prevents formal comparison of subtype distributions. In acute symptomatic AF, recent onset, absence of heart failure, smaller left atrial size, and other presentation features have been associated with spontaneous conversion, underscoring why precise temporal phenotyping matters for rhythm-management decisions [24].

The anticoagulant data should be interpreted as inpatient medication administration, not chronic stroke-prevention quality. The 2024 ESC guideline favours CHA_2_DS_2_-VA and individualized reassessment, while HAS-BLED is intended to identify modifiable bleeding factors rather than justify withholding anticoagulation [21]. Latin American GARFIELD-AF data have demonstrated substantial between-country variation in long-term antithrombotic treatment [25], but the present study lacks baseline and discharge prescriptions, contraindications, procedures, valvular context, dosing, and the indication for enoxaparin. It therefore cannot determine whether non-administration represented appropriate temporary management or undertreatment.

Altitude is best regarded here as a characteristic of the hospital setting. Hypobaric hypoxia can influence sympathetic activity, pulmonary vascular physiology, and haemostasis [22,26,27,28] but this study measured neither individual altitude exposure nor oxygenation or haematologic adaptation and had no lower-altitude comparator. The mean estimated systolic pulmonary artery pressure cannot be attributed to altitude because advanced age and coexisting cardiac or pulmonary disease offer competing explanations.

The five in-hospital deaths provide a useful absolute outcome estimate but cannot support stable associations or prognostic claims. The supplemental univariable comparisons showed several nominal raw *p* values, but none remained significant after false-discovery-rate correction. The wide confidence intervals and sparse cells demonstrate the instability expected with this event count; these comparisons are therefore hypothesis-generating only, and substantially larger samples with longer follow-up would be required for adjusted prognosis modelling [29].

From a clinical perspective, structured AF pathways could improve documentation of diagnostic confirmation, temporal pattern, stroke-risk reassessment, modifiable bleeding factors, medication reconciliation, reasons for treatment interruption, and discharge therapy. These process measures would allow future studies to distinguish justified inpatient decisions from persistent gaps in long-term stroke prevention.

The principal contribution of this study is a transparent description of AF managed in an underrepresented Andean hospital context. Multicentre prospective work should include ECG adjudication, AF status at admission, individual residential altitude and duration, oxygen saturation, hemoglobin/haematocrit, lower-altitude comparators, detailed anticoagulant indications and dosing, and longitudinal outcomes.

## 5. Limitations

This study is limited by its retrospective, single-centre design, reliance on medical-record documentation, and predominantly private-sector and mestizo cohort. It may therefore be affected by selection and information bias and may not represent rural, public-sector, Indigenous, or lower-complexity populations. The fixed 2021–2023 window reflects the closed dataset specified for the approved retrospective protocol and does not capture subsequent practice changes.

Case ascertainment used ICD-10 I48 plus clinician documentation; ECG tracings were not systematically re-adjudicated, complete exclusion of atrial flutter was not possible when documentation was ambiguous, and AF could not be consistently classified as the primary, secondary, or incidental diagnosis. “First-diagnosed” was not a guideline-standard temporal subtype. These factors create potential diagnostic and classification misclassification.

Individual altitude exposure, duration of residence, oxygenation, hemoglobin/haematocrit, and a lower-altitude comparator were unavailable, precluding altitude-related inference. Formal CHA_2_DS_2_-VASc and HAS-BLED scores were missing in 39 patients (16.4%), and CHA_2_DS_2_-VA could not be reconstructed from the aggregate export. Baseline and discharge anticoagulation, contraindications, procedures, valvular context, enoxaparin indication, and direct oral anticoagulant dose were incomplete. Measurement-specific denominators for continuous echocardiographic variables were not retained. Finally, only five deaths occurred. Although univariable comparisons are provided in Supplementary Table S1 for exploratory completeness, none remained significant after false-discovery-rate correction, no multivariable model was fitted, and no prognostic inference is warranted.

## 6. Conclusions

Adults with AF treated at this tertiary hospital were older and commonly had hypertension, heart failure, and high chart-recorded thromboembolic-risk scores. Any anticoagulant was administered to most patients during hospitalization, but the available data cannot establish the appropriateness or continuity of chronic stroke-prevention therapy. Hospital altitude is contextual and does not define an altitude-related AF phenotype. Exploratory survival-status comparisons did not remain significant after false-discovery-rate correction and do not support prognostic inference. Prospective multicentre studies with adjudicated diagnosis, contemporary CHA_2_DS_2_-VA assessment, detailed longitudinal treatment data, individual hypoxia-related measurements, and lower-altitude comparators are needed.

## Figures and Tables

**Table 1:** Demographic, anthropometric, and behavioural characteristics of patients with atrial fibrillation.

Characteristic	Category	Value
Age, years	—	77.9 ± 13.3
Sex	Female	107 (45.0)
	Male	131 (55.0)
Residence	Ecuador	228 (95.8)
	Outside Ecuador	10 (4.2)
Recorded ethnicity	Mestizo	231 (97.1)
	White	5 (2.1)
	Other	2 (0.8)
Body-mass index, kg/m^2^	—	27.6 ± 4.5
Tobacco use	Yes	44 (18.5)
Alcohol use	Yes	5 (2.1)
Chart-documented regular physical activity	Yes	41 (17.2)
Tobacco exposure among users, pack-years	—	20.1 ± 15.1
Exercise frequency among active patients, sessions/week	—	4.9 ± 2.0

Values are n (%) or mean ± SD. Regular physical activity denotes habitual activity explicitly documented in the medical record; no standardized intensity or duration threshold was available.

**Table 2:** Clinical comorbidities and cardiovascular history of patients with atrial fibrillation.

Condition/History	n (%)
Hypertension	138 (58.0)
Heart failure	54 (22.7)
Hypothyroidism	45 (18.9)
Type 2 diabetes mellitus	36 (15.1)
Dyslipidaemia	34 (14.3)
Obesity	32 (13.4)
Previous myocardial infarction	24 (10.1)
Previous cardiac surgery	19 (8.0)
Chronic kidney disease	16 (6.7)
Previous ischaemic stroke	14 (5.9)
Coronary stent	14 (5.9)
Sleep apnoea	9 (3.8)
Dialysis	4 (1.7)

Values are n (%). Only positive categories are presented.

**Table 3:** Atrial fibrillation presentation and in-hospital management of patients with atrial fibrillation.

Characteristic	Value
Symptomatic AF	121 (50.8)
Family history of AF	14 (5.9)
Not in sinus rhythm at admission	157 (66.0)
NYHA functional class II	98 (41.2)
Pharmacological AF treatment documented	182 (76.5)
Length of stay, days	4.9 ± 4.6
In-hospital death	5 (2.1)

Values are n (%) or mean ± SD. AF, atrial fibrillation; NYHA, New York Heart Association.

**Table 4:** Chart-documented thromboembolic and bleeding-risk scores in patients with atrial fibrillation.

Score	Patients Scored, n	Mean ± SD	Median (IQR)	Threshold Summary, n (%)
CHA_2_DS_2_-VASc	199	3.3 ± 1.4	3 (2–4)	≥2: 185 (93.0)
HAS-BLED	199	2.3 ± 1.1	2 (2–3)	≥3: 65 (32.7)

IQR, interquartile range. Scores were recorded by the treating cardiology team and available in 199 of 238 patients. CHA_2_DS_2_-VASc is reported historically and was not recalculated as CHA_2_DS_2_-VA.

**Table 5:** Echocardiographic findings in patients with atrial fibrillation.

Variable	Finding
Left-ventricular ejection fraction, %	59.2 ± 11.7
Left atrium dilated	99/233 (42.5)
Left-atrial volume index, mL/m^2^	51.9 ± 25.3
Estimated systolic pulmonary artery pressure, mmHg	41.9 ± 16.8
E/e′ ratio	12.5 ± 6.0
Mitral valve abnormality	81/238 (34.0)
Aortic valve abnormality	50/238 (21.0)
Tricuspid valve abnormality	104/238 (43.7)
Pulmonary valve abnormality	34/238 (14.3)
Wall-motion abnormality	25/238 (10.5)

Continuous variables are mean ± SD and categorical variables are n/N (%). Measurement-specific denominators for continuous echocardiographic variables were not retained in the de-identified aggregate export; estimates therefore reflect available observations.

## Data Availability

The data used for this research can be requested from the corresponding author.

## Supplementary Materials

The following supporting information can be downloaded at: https://www.mdpi.com/article/10.3390/medicina62091736/s1: Table S1. Exploratory univariable comparisons between survivors and non-survivors.

## Abbreviations

The following abbreviations are used in this manuscript:

AF	Atrial fibrillation
BMI	Body mass index
MI	Myocardial Infarction
HF	Heart Failure
HTN	Hypertension
T2DM	Type 2 Diabetes Mellitus
CKD	Chronic Kidney Disease
ECG	electrocardiogram
