# Supplementary material for: Clinical and Epidemiological Profile of Atrial Fibrillation in a High-Altitude Tertiary-Care Setting
Source: Medicina (Kaunas). 2026 Sep 9;62(9):1736. doi: 10.3390/medicina62091736 (PMC13609408; doi:10.3390/medicina62091736)
Supplement: Supplementary file 1 [file medicina-62-01736-s001.zip › medicina-4470890-supplementary.pdf]

Supplementary Table S1. Exploratory univariable comparisons between survivors and non-survivors.

**Continuous variables**					
**Variable**	**Survivors (n = 233)**	**Non-survivors (n = 5)**	**Mean difference (95% CI)**	**Raw p**	**FDR-adjusted q**
Age, years	77.6 ± 13.2	90.2 ± 8.8	12.6 (1.8 to 23.4)	0.031	0.321
Height, cm	161.1 ± 11.7	158.6 ± 10.5	−2.5 (−15.4 to 10.4)	0.626	1.000
Weight, kg	72.2 ± 16.8	72.0 ± 14.4	−0.2 (−17.9 to 17.5)	0.977	1.000
Body-mass index, kg/m^2	27.8 ± 4.4	19.8 ± 2.8	−8.0 (−11.4 to −4.6)	0.002	0.074
**Categorical variables**					
**Variable, positive category**	**Survivors, n/N (%)**	**Non-survivors, n/N (%)**	**Odds ratio (95% CI)**	**Fisher exact p**	**FDR-adjusted q**
Female sex	104/233 (44.6)	3/5 (60.0)	1.86 (0.21–22.60)	0.660	1.000
Residence outside Ecuador	9/233 (3.9)	1/5 (20.0)	6.12 (0.11–71.03)	0.195	0.817
Mestizo ethnicity	226/233 (97.0)	5/5 (100.0)	∞ (0.02–∞)	1.000	1.000
Tobacco use	42/233 (18.0)	2/5 (40.0)	3.01 (0.24–27.16)	0.231	0.817
Alcohol use	5/233 (2.1)	0/5 (0.0)	0.00 (0.00–63.84)	1.000	1.000
Regular physical activity	40/233 (17.2)	1/5 (20.0)	1.21 (0.02–12.61)	1.000	1.000
Previous cardiac surgery	18/233 (7.7)	1/5 (20.0)	2.97 (0.06–32.14)	0.343	0.885
Previous myocardial infarction	23/233 (9.9)	1/5 (20.0)	2.27 (0.04–24.27)	0.415	0.919
Previous STEMI	4/233 (1.7)	1/5 (20.0)	13.77 (0.23–195.04)	0.102	0.630
Coronary stent	13/233 (5.6)	1/5 (20.0)	4.18 (0.08–46.54)	0.263	0.817
Heart failure	52/233 (22.3)	2/5 (40.0)	2.31 (0.19–20.73)	0.318	0.885
Hypertension	134/233 (57.5)	4/5 (80.0)	2.94 (0.29–146.98)	0.402	0.919
Previous ischaemic stroke	13/233 (5.6)	1/5 (20.0)	4.18 (0.08–46.54)	0.263	0.817
Type 2 diabetes mellitus	36/233 (15.5)	0/5 (0.0)	0.00 (0.00–6.21)	1.000	1.000
Dyslipidaemia	33/233 (14.2)	1/5 (20.0)	1.51 (0.03–15.91)	0.541	1.000
Hypothyroidism	43/233 (18.5)	2/5 (40.0)	2.93 (0.24–26.38)	0.239	0.817
Obesity	32/233 (13.7)	0/5 (0.0)	0.00 (0.00–7.16)	1.000	1.000
Chronic kidney disease	16/233 (6.9)	0/5 (0.0)	0.00 (0.00–15.99)	1.000	1.000
Sleep apnoea	9/233 (3.9)	0/5 (0.0)	0.00 (0.00–31.05)	1.000	1.000
No ECG documented at admission	29/233 (12.4)	3/5 (60.0)	10.36 (1.14–128.98)	0.018	0.285
No anticoagulant administered during hospitalization	40/233 (17.2)	3/5 (60.0)	7.15 (0.79–88.16)	0.042	0.328
Left-atrial dilation	97/228 (42.5)	2/5 (40.0)	0.90 (0.07–8.02)	1.000	1.000
Mitral valve abnormality	79/233 (33.9)	2/5 (40.0)	1.30 (0.11–11.57)	1.000	1.000
Aortic valve abnormality	49/233 (21.0)	1/5 (20.0)	0.94 (0.02–9.77)	1.000	1.000
Tricuspid valve abnormality	102/233 (43.8)	2/5 (40.0)	0.86 (0.07–7.62)	1.000	1.000
Pulmonary valve abnormality	34/233 (14.6)	0/5 (0.0)	0.00 (0.00–6.66)	1.000	1.000
Wall-motion abnormality	25/233 (10.7)	0/5 (0.0)	0.00 (0.00–9.57)	1.000	1.000

Note: For continuous variables values are mean ± SD. Mean differences are calculated as non-survivors minus survivors. Confidence intervals and raw p values were obtained using Welch's t test. For categorical variables positive categories are reported as n/N (%). Odds ratios are conditional maximum-likelihood estimates with exact 95% confidence intervals; raw p values are two-sided Fisher exact p values. FDR-adjusted q values were calculated using the Benjamini–Hochberg procedure across all 31 comparisons in Panels A and B. Missing observations were handled by available-case analysis without imputation. Because only five deaths occurred, all comparisons are post hoc and hypothesis-generating; no multivariable model was fitted. CI, confidence interval; ECG, electrocardiogram; FDR, false discovery rate; SD, standard deviation; STEMI, ST-elevation myocardial infarction.
